# Virtual Sensors for Designing Irrigation Controllers in Greenhouses

**DOI:** 10.3390/s121115244

**Published:** 2012-11-08

**Authors:** Jorge Antonio Sánchez, Francisco Rodríguez, José Luis Guzmán, Manuel R Arahal

**Affiliations:** 1 Ingeniería de Sistemas y Automática, Departamento de Lenguajes y Computación, The Agrifood Campus of International Excellence (ceiA3), Universidad de Almería, Ctra de la Playa s/n., Almería 04120, Spain; E-Mails: frrodrig@ual.es (F.R.); joguzman@ual.es (J.L.G.); 2 Departamento de Ingeniería de Sistemas y Automática, Escuela Superior de Ingenieros, Universidad de Sevilla, Camino de los Descubrimientos s/n, Sevilla 41092, Spain; E-Mail: arahal@esi.us.es

**Keywords:** virtual sensor, transpiration, nonlinear model, micro-lysimeter

## Abstract

Monitoring the greenhouse transpiration for control purposes is currently a difficult task. The absence of affordable sensors that provide continuous transpiration measurements motivates the use of estimators. In the case of tomato crops, the availability of estimators allows the design of automatic fertirrigation (irrigation + fertilization) schemes in greenhouses, minimizing the dispensed water while fulfilling crop needs. This paper shows how system identification techniques can be applied to obtain nonlinear virtual sensors for estimating transpiration. The greenhouse used for this study is equipped with a microlysimeter, which allows one to continuously sample the transpiration values. While the microlysimeter is an advantageous piece of equipment for research, it is also expensive and requires maintenance. This paper presents the design and development of a virtual sensor to model the crop transpiration, hence avoiding the use of this kind of expensive sensor. The resulting virtual sensor is obtained by dynamical system identification techniques based on regressors taken from variables typically found in a greenhouse, such as global radiation and vapor pressure deficit. The virtual sensor is thus based on empirical data. In this paper, some effort has been made to eliminate some problems associated with grey-box models: advance phenomenon and overestimation. The results are tested with real data and compared with other approaches. Better results are obtained with the use of nonlinear Black-box virtual sensors. This sensor is based on global radiation and vapor pressure deficit (VPD) measurements. Predictive results for the three models are developed for comparative purposes.

## 1. Introduction

Crop growth is primarily determined by climatic variables of the environment and the amount of water and fertilizers applied through irrigation. Therefore, controlling these variables allows for control of the growth. The greenhouse environment is ideal for farming because these variables can be manipulated to achieve optimal growth and plant development. All crops need solar radiation, CO2, water, and nutrients to produce biomass (roots, stems, leaves, and fruits) through the process of photosynthesis. During this process, and when the leaves stomata are opened to capture the CO2, the plant emits water vapor through the transpiration process. This becomes a cost that the crop must make to produce dry matter. Moreover, water is lost through evaporation from the soil. The sum of these water losses is known as evapotranspiration. The losses must be compensated through irrigation. Besides, it has been demonstrated in padded greenhouses with soil covered with plastic blankets that the amount of evaporation is negligible. This happens when dealing with hydroponic cultivations [1]. According to this, water should be applied in precise amounts to cover only water losses due to crop transpiration. Excess water would mean an excessive washing out of fertilizers. In turn, it could lead to contamination of the subterranean water, or the flooding of the substratum or radicular asphyxiation. Otherwise, a hydric deficit may be provoked if irrigation does not provide enough water. This can lead to a decrease in production and can even be dangerous for the crop growth. Hence, automatic irrigation control systems are fundamental tools to supply water to the culture in the required amount and frequency. Moreover, as water is a limited resource in many agricultural areas, optimizing productivity through efficient and adequate irrigation is a basic objective. In order to design a good automatic irrigation system, the following questions must be answered: what should the frequency of the irrigations be, and how much water should be applied in the irrigation? To answer these questions, it is necessary to know how much water should be applied to replenish the losses due to the transpiration during the plant's respiration.

Measuring the water lost by transpiration is a way of obtaining the plant's water demand. This estimation of transpiration in different species grown in greenhouses has been developed, e.g., by Baille *et al.* [2] for ornamentals, Stanghellini [1], Jemaa [3], Boulard [4] and Baille [2] for tomatoes, Montero *et al.* [5] for geranium crops, Medrano *et al.* [6] for cucumbers, Suay *et al.* [7] for rose cultivation, Voogt *et al.* [8] for chrysanthemums, and Schmidt and Exarchou [9] for gerbera pots, among others.

In most of these works, the microlysimeter became the basic measurement device to record the water losses in crops, subtracting the water content in an instant (*t*) by the water content in another instant (*t* − 1). However, on many occasions, the measurements were not continuous due to the irrigation process or during the water drainage. Furthermore, it is seldom used by farmers since this device is expensive to acquire and to maintain. From an operational point of view, it is important to find alternatives to this irrigation system gadget. Thus, virtual sensors based on transpiration become a good option to reduce total system cost, especially in the agriculture sector where profit margins are so narrow. Such virtual sensors must be based on sensors that are typically installed in greenhouses for climate control (temperature, humidity, and solar radiation), thereby reducing the installations costs.

Virtual sensors become a very efficient and powerful tool that has been successfully used in other fields [10–12]. These sensors utilize models in order to estimate features from low-cost measurements. Ideally, the virtual sensors should be simple and obtainable from the collected data. It should not require extensive training. Virtual sensors are useful in replacing physical sensors, thus reducing hardware redundancy and acquisition cost, or as part of the fault detection methodologies by having their output compared with that of a corresponding actual sensor. Virtual sensors may be developed based on mathematical models obtained directly from the Physics of the system and first principles. In many cases, such mathematical models are unavailable, or their exact parameter values are unknown, or they are too complicated to be used. For this reason, the development of virtual sensors often has to be based on system identification [13].

The purpose of the current study is to develop a virtual sensor to infer transpiration from other easily measured variables. For this purpose, the use of the microlysimeter as the sensor to calculate the transpiration must be substituted, due to its high cost in acquisition and maintenance. In this paper, the development of the virtual sensor for transpiration makes use of different techniques for data preprocessing, including the selection of variables, the construction of appropriate training, the selection of test sets, the final validation and the performance assessment. The resulting virtual sensor has been validated and compared with real data and with other virtual sensors in the literature, providing promising results.

The paper is organized as follows: Section 2 shows a background of different sensors used to take transpiration measurements. The different virtual sensors are shown in the Section 3. Section 4 gives an overview of the greenhouse where the experiments were performed, and its main characteristics, as well as the collected experimental data. The main results and discussions are summarized in Section 5. Finally, the major conclusions are drawn.

## 2. Crop Transpiration

**Figure 1.**
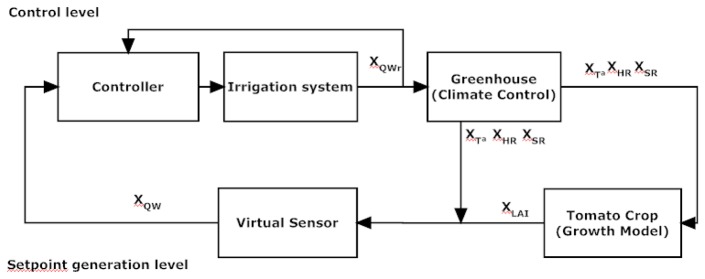
Irrigation control algorithm.

The irrigation control systems are essential tools to provide water to the crop in the required amount and frequency. Moreover, water is a limiting resource in many agricultural areas. In such places, it should be a basic objective to optimize their management and productivity through adequate and efficient irrigation. The proposed control algorithm design (Figure 1) is a hierarchical control system, consisting of two levels:

- The control level uses an event-based PI controller [14], to control when a certain event occurs, either by time, by variation of a particular climatic variable such as radiation *V_S_R__*), or by a particular state crop. A PI controller is used to achieve the setpoint of water supply (*X_Q_Wr*) as it considers the top layer of the architecture.
- The setpoint generation level is based on the greenhouse climate conditions, including: (1) vapor pressure deficit (VPD, *V_V PD_*), which is a function of the temperature and the relative humidity, (2) global radiation, and (3) the state of the crop, measured through the Leaf Area Index (LAI, *X_LAI_*). The setpoint is fixed by the user and is defined as the crop transpiration accumulated (*X_ET_*) until an irrigation event occurs (*X_Q_W*). This event could be a determined transpiration accumulated setpoint or other predetermined conditions.

The virtual sensor accumulates the amount of water lost by transpiration from the plant's last irrigation. This measurement is compared with the fixed amount that activates irrigation (setpoint). The irrigation starts when this fixed value is exceeded, and finishes as soon as the amount of water lost has been replenished.

A virtual sensor requires an accurate calibration and validation of the instantaneous transpiration measurements at each sampling instant. The measurement of transpiration includes direct (observation, porometer, lysimeter, *etc.*), and indirect methods (water budget, energy balance, *etc.*). The indirect methods are more complicated for prediction and verification of the transpiration values because of the meteorological factors involved. The transpiration data can be collected through direct methods such as:

- The *porometer* [15] allows the determination of the leaf conductivity as an index of the stomatal opening and the closing of stomata. It measures the flow of gases or diffusion that takes place through the stomata. The latest porometers allow computerized records.
- The *bag method* [16] collects the water transpired by introducing a branch in a clear plastic bag. The transpired water condenses inside the bag. The total water lost by transpiration corresponds with the weight of the placed water. The time between measurements is undefined, as it depends on the water collected.
- In the *cobalt chloride* method [17], the transpiration is indicated by a color change of a piece of filter paper impregnated with a 3% solution of cobalt chloride. It is applied on a leaf and held in place with a clip. It is blue when dry and pink when wet. The speed at which the paper changes color is an indication of the rate of transpiration. This method can be used to measure the relative rates of transpiration of different species.
- The *microlysimeter* [1,4] is used in plants growing in pots completely closed. The plants are first weighed before measuring, and then they are weighed again at convenient time intervals. Soil evaporation is avoided by covering it with waterproof material. This method can be used with small plants and crops in soilless culture. The results are expressed in grams or milliliters of water transpired per leaf area per unit time.

The microlysimeter is the basic measurement device used to continuously record the water losses in crops. Contrarily, the rest of the described sensors take measurements at intervals as they modify the working conditions of the plants. The porometer and microlysimeter are expensive to acquire and maintain, and are difficult for the farmers to manage.

## 3. Virtual Sensors

This section is devoted to describing the main features of three different types of virtual sensors for transpiration designed to replace the microlysimeter in the automatic irrigation system. The aim of such virtual sensors is to substitute the expensive microlysimeter in measuring the transpiration and controlling the irrigation.

**Figure 2.**
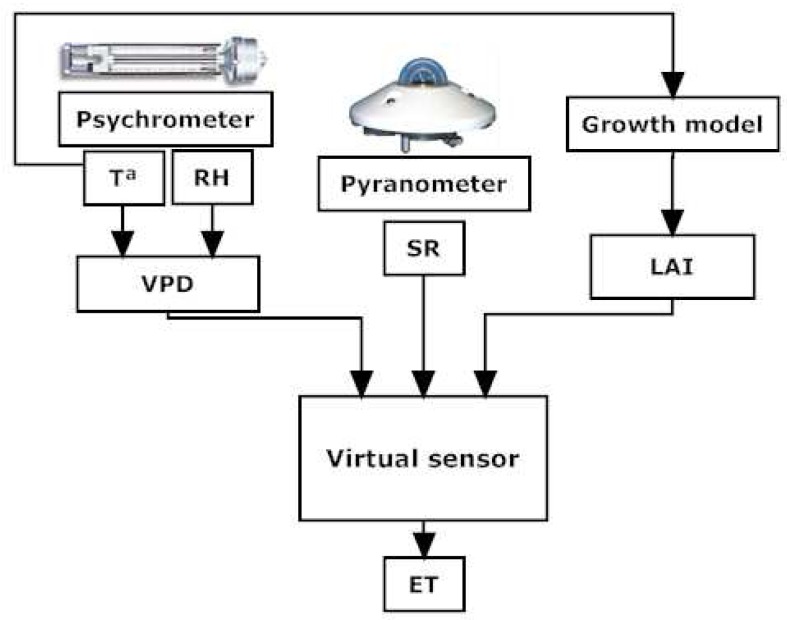
Virtual sensor scheme.

Figure 2 shows the input variables of a virtual sensor for crop transpiration: (1) global (solar) radiation, (2) Vapor Pressure Deficit (VPD), which is a function of the temperature (T) and the relative humidity (RH), and (3) Leaf Area Index (LAI). These variables are measured by two different sensors typically installed in commercial greenhouses: a psychrometer for the temperature and the relative humidity (VPD), and a pyranometer for the solar radiation.

The transpiration model needs to calculate the LAI. The LAI measurements are taken in a noncontinuous way. In this paper, a simplified TOMGRO model [18] adapted to the Mediterranean conditions is utilized to estimate the LAI. The simplified TOMGRO model needs the temperature as the input.

Based on the architecture of Figure 2, three different virtual sensors are considered in this paper. The first one is based on a pseudo-physical structure. The others have a more empirical structure with linear and nonlinear behaviors, respectively. All three kinds are described in the following.

### 3.1. Pseudo-Physical (Grey-Box) Virtual Sensor

Most transpiration estimators are based on the Penman–Monteith equation. In 1948, Penman derived an equation that combined the energy balance and the convective transport of vapor. Afterwards, this model was adapted by Monteith to estimate actual evapotranspiration from plants [19]. This equation essentially combines the equation for heat transfer between the crop and the mass of the surrounding air. In this way, various authors have obtained new formulations without satisfactory results for various crops. The tomato evapotranspiration studies of [20] have as a main drawback the estimation of the leaf stomatal and aerodynamical resistances. Jolliet and Bailey [15] concluded that a layer model proposed in [1] predicts accurately the transpiration in tomatoes. These authors observed the tomato crop transpiration (*X_ET_*) increases linearly with the radiation (*V_S_R__*), the vapor pressure deficit (*V_V PD_*), and the wind speed inside the greenhouse. They also pointed out that transpiration can be regulated by not only humidity but also radiation and wind speed. These same authors simplified the Penman–Monteith equation [21]. to describe the transpiration (*X_ET_*) as a process based on two main variables: the solar radiation (*V_S_R__*) arriving at a particular depth in the canopy plant, and the vapor pressure deficit *V_V PD_* Equation (1). The reduced virtual sensor is shown in the following equation:

(1) $$C_{\lambda }X_{\text{ET}}=V_{S_{R}}C_{A}+V_{V\;\text{PD}}C_{B}$$

where the coefficients *C_A_* and *C_B_* are the parameters dependent on the crop.

In [2], it was observed that the coefficient *C_B_* increases with the *X_LAI_*, and, furthermore, the coefficient adopted different values during the day due to oscillations in stomatal resistance Equation (2). These daily oscillations was corrected with two different parameters for diurnal (*C_B_D__*) and nocturnal (*C_B_N__*) periods.

(2) $$C_{\lambda }X_{\text{ET}}=e^{\left(-C_{k}X_{\text{LAI}}\right)}V_{G_{R}}C_{A}+V_{\text{DPV}}X_{\text{LAI}}C_{B}$$

where *X_ET_* is the crop evapotranspiration (*gm*^−2^*min*^−1^), *C_λ_* is the latent heat of evaporation (*kg*/°*C*), *C_k_* is the light extinction coefficient for crops (it is related to the leaf inclination angle and the leaf arrangement with regard to the Leaf Area Index, and provides an indication of the plant's efficiency on intercepting the solar radiation), *X_LAI_* is the leaf area index (*m*^2^*m*^−2^), *V_V PD_* is the vapor pressure deficit (*KPa*), and *V_G_R__* is the global radiation reaching the crop (*Wm*^−2^). The coefficients *C_A_* (unitless) and *C_B_* (*kgm*^−2^*h*^−1^*kPa* ^−1^) are constants dependent on the crop. To obtain more reliable results of the virtual sensor, the parameter *C_B_* is obtained for diurnal (*C_B_D__*) and nocturnal (*C_B_N__*) periods through calibration.

### 3.2. Black-Box (Empirical) Virtual Sensors

The identification system tries to solve the problem of constructing mathematical models of dynamic systems based on the data they observe [22]: inputs *u*(*t*) and outputs *y*(*t*). The goal is to infer the relationship between sampled outputs and inputs. The identification process was carried out based on prior knowledge of dynamic behavior of gases [14] and the behavior of transpiration ([1,2,6]). VPD and global radiation was selected as climatic inputs and the LAI as crop growth input.

This method is used with the objective of knowing how the dynamics of the systems work and responding to some of the questions raised while drawing up this document.

#### 3.2.1. Linear Black-Box Virtual Sensors

The parametric virtual sensors (or black box) are diagrams capable of representing any system without having any knowledge about the physical process dynamics. Parametric virtual sensors are not obtained (at least not completely) from the application of physical laws. These virtual sensors are constructed using observations carried out on the system so as to select a concrete value of the parameters. This value is chosen in such a way that the virtual sensor can accommodate the results to the acquired data. This process is called identification.

The adjustment of the parameters is the simplest part of the problem of identification [23]. Online identification concerns an algorithm that efficiently uses the measured information when it is obtained from the plant in real time. In this way, it is possible to detect the changes in the dynamics of the system and adjust the virtual sensor conveniently. Under some circumstances, these methods can be rather simple (e.g., the method of minimal recurrent squares developed in this section).

**Figure 3.**
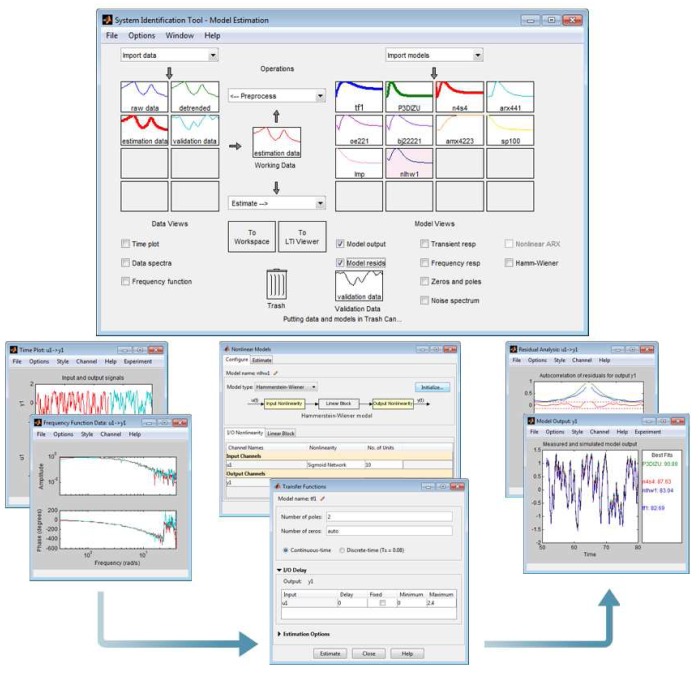
Graphical user interface of “ident”, a part of the System identification Matlab-Toolbox® [24].

The black-box virtual sensors are developed using a system identification technique shown in [23]. The virtual sensors family contains 32 possible formulations based on Equation (3). To obtain each one of the structures, it is necessary to determine the polynomial order as well as the coefficient of the numerator and the denominator for each transfer function [23]. The effects to the inputs, output, and the disturbances are defined as:

(3) $$A(z)y(t)=\frac{B(z)}{F(z)}u(t-nk)+\frac{C(z)}{D(z)}e(t)$$

where, *y*(*t*) is the transpiration, *u*(*t*) is the different input variables, and *e*(*t*) is the estimation error. *A*(*z*), *B*(*z*), *C*(*z*), *D*(*z*), *E*(*z*), and *F*(*z*) represent the polynomials that define the output (transpiration), inputs, and the estimation error. The order of the polynomial equations is defined by regressors, where *na* outlines the outputs, *nb* is the order of the input, and *nk* is the delay of the radiation solar (*nk_V_G_R___*) and the vapor pressure deficit (*nk_V_V PD__*). System Identification Matlab's Toolbox [24] was used for the identification process. The ARX, ARMAX, OUTPUT ERROR, BOX JENKINS, and FIR formulations were tried out. The differences among these virtual sensors are the way in which the inputs, outputs and disturbances are defined with parametric equations. The System Identification Toolbox of Matlab^(^*^R^*^)^ software was used to obtain the virtual sensor. Figure 3 shows the main interface of the Toolbox and the whole process to obtain a virtual sensor. The top of the figure displays the calibration and validation process interface, and the bottom shows the output analysis process. The toolbox allows to process data, estimate the parameters of different types of structures, and validate virtual sensors using different strategies. For the identification process, only the data from the experiments are required. The data can be handled in the time domain or frequency domain, and the experiments can have one or multiple inputs and/or outputs [25].

#### 3.2.2. Nonlinear Black-Box Virtual Sensors

**Figure 4.**
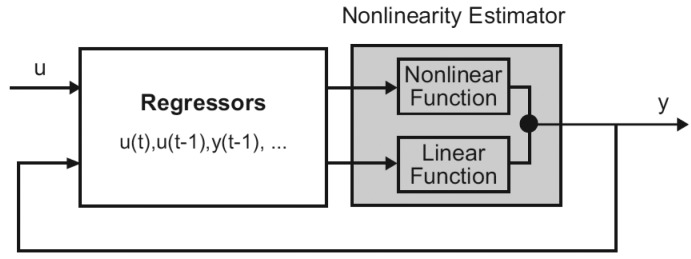
The Nonlinear ARX computes the output y in two stages [24]. u(t) are the model inputs, and y(t) are the model outputs.

A nonlinear component was added to the transpiration virtual sensor to get better fitting. This component is introduced as result of the strong nonlinear behavior in the system inputs. Moreover, these nonlinearities add complexity to the virtual sensor. This increase in complexity is not always translated into higher performance. In System Identification the mathematical relationships between the system's inputs *u*(*t*), and outputs *y*(*t*) can be computed. Such outputs, inputs, and nonlinearities are introduced in an *ad hoc* form, relying on *a priori* knowledge about the system. An important step in system identification is to choose a structure, and generally start testing the simpler structures, and lower order. The first structure tested, and in the end chosen as virtual sensors, was the nonlinear ARX (4). Also the Hammerstein–Wiener virtual sensor was tried out, which are very useful in the case of the nonlinearities affect to sensors, and actuators, such as dead zones or saturation [24]. On the other hand, Nonlinear ARX (NonARX) is more flexible [24]. The general structure for Nonlinear ARX virtual sensor is [26]:

(4) y(t)=f(y(t−1),y(t−2),y(t−3),…,u(t),u(t−1),u(t−2),..)

where *y(t)* is the output variable in *t* time; *u* and *y* are the different input and output variables (regressors); and *f* is the nonlinear function. The current transpiration value is predicted as a weighted sum of past values, and current and past inputs values. With such information the equation becomes:

(5) $$y(t)=[a_{1},a_{2},\ldots ,a_{\text{na}},b_{1},b_{2},\ldots ,b_{\text{nb}}]{\left[y(t-1),y(t-2),\ldots ,y(t-\text{na}),u(t),u(t-1),\ldots ,u(t-\text{nb}-1)\right]}^{T}$$

where *y*(*t* − 1), *y*(*t* − 2), …, *y*(*t* − *na*), *u*(*t*), *u*(*t* − 1), …, *u*(*t* − *nb* − 1 are the regressors, the so-called delayed inputs and outputs. Nonlinear ARX regressors can be both delayed input–output variables and more complex nonlinear expressions of delayed variables. The nonlinearity estimator block maps the regressors to the virtual sensor output using a combination of nonlinear and linear functions (Figure 4).

Available nonlinearity estimators can be selected from a canopy of different structures such as neural networks, tree-partition networks, wavelet networks and piecewise polynomial approximation. The nonlinearity estimator block can include linear and nonlinear blocks in parallel [24].

## 4. Greenhouse Environment

**Figure 5.**
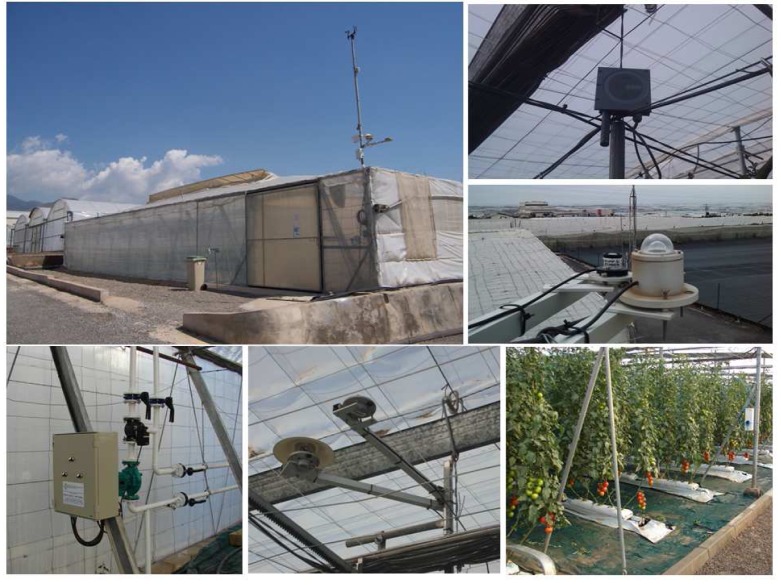
Greenhouse facilities used for the experiences performed in this work. From left to right and from top to bottom on the figure: Greenhouse, CO_2_ sensor, Solar and PAR radiation, Heating system, Solar and PAR radiation inside the greenhouse, and the tomato crop lines.

The research data used in this work have been obtained from greenhouses located in the Experimental Station of Cajamar Foundation, in El Ejido, in the province of Almeria, Spain (2°43′W, 36°48′N, and 151 m elevation). The crops grows in a multispan “Parral-type” greenhouse (Figure 5); [18]. The greenhouse has a surface of 877 m^2^ (37.8 × 23.2 m), polyethylene cover, automated ventilation [27] with lateral windows in the northern and southern walls, flap roof window in each span, mesh-protected anti-trips “bionet” of 20 × 10 thickness, and night heating applied with a 95 kW hot air heater that is programmed to maintain the minimum temperature above 14 °C. The greenhouse orientation is east–west with the crop rows aligned north–south. Cropping conditions and crop management are very similar to those in commercial greenhouses.

Climatic parameters are continuously monitored within the greenhouse. Outside the greenhouse, a meteorological station was installed, in which air temperature, relative humidity, solar and photosynthetic active radiation (PAR), rain detector, wind direction, and velocity measurements were taken. The cover temperature sensors were located on the faces oriented to the east (two sensors), and west (two sensors).

During the experiments, the inside climate variables were also taken, among which stand out: air temperature, and relative humidity with a ventilated psychrometer (model MTH-A1, ITC, Almeria, Spain), solar radiation with a pyranometer (model MRG-1P, ITC, Almeria, Spain), and Photosynthetic active radiation (PAR) with a silicon sensor (PAR Lite, Kipp–Zonnen, Delft, The Netherlands). Among all the climate sensors installed in the greenhouse, only solar radiation and psychrometer was used for the transpiration virtual sensors.

The daylight air temperature and humidity are controlled by the top and side windows through the PI controller [28]. Potentiometers allow for knowing the window's position in each control instant. The night air temperature and humidity is controlled by the windows and the heating system [28]. Setpoints of both systems are established at 24 °C [27], and 14 °C for the ventilation and heating, respectively. All the actuators are driven by relays designed for this task.

All climatic data was recorded every minute with a personal computer. The acquisition system is formed by two different National Instrument Compact-Fieldpoints®connected through Ethernet protocol.

For the growth model, it was necessary to know the evolution of leaf area index. It was determined through the leaf area measurements of each plant removed for biomass task, the pruning, and deleafing were also taken into account. The biomass was made up of a destructive sampling of five randomly selected plants every 21 days, duration accorded in the research protocol. The choice of 21 days is twofold: first, it was the sufficient amount of time to find growth differences; and second, it helped to avoid the elimination of too much vegetal stuff in the greenhouse which could end in a modification in the climate or transpiration measurements.

The biomass process was measured against: number of nodes, leaf area, number of fruits per bunch, fresh and dry weight of leaves, stem, and fruits. The plant material and fruits were introduced into a drying oven where they remained for 24–48 h (depending on the phenological state) at a temperature of 65 °C. Based on this, the dry matter of leaves, stems, and fruits was determined by analytical balance. The matter of leaves and secondary stems pruning came from the selected plants for biomass while kept in production; once removed from the plant, the fresh and dry weight was taken, such as the biomass. In the case of pruning, stems and leaves are measured separately. Both the bare and the pruning are carried out for the leaf area index measures, executed, as in the biomass, through electronic planimeter (Delta-T Devices Ltd).

**Figure 6.**
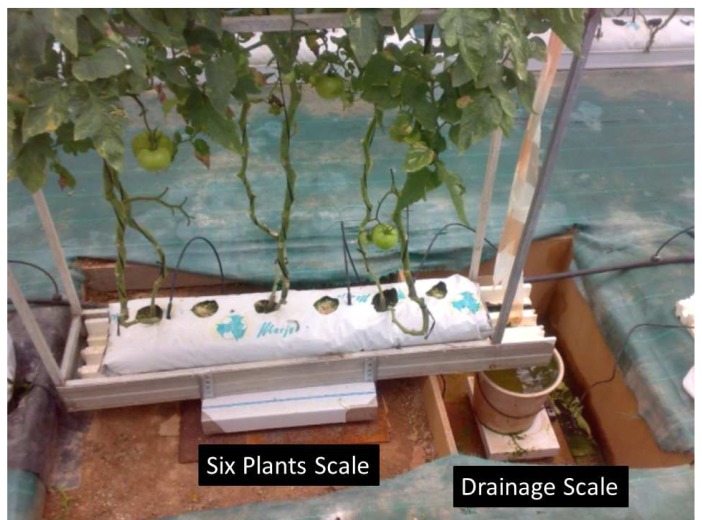
Lysimeter installed in the greenhouse for the transpiration calculation.

Microlysimeter was the system chosen to take the transpiration measurement in the present paper ([18]; Figure 6). The device consists in two electronic weighing scales connected to a personal computer. The first (150 kg ± 1 g, Sartorius) records the weight of a bag with six plants, and a support structure. The second weighing scale (20 kg ± 0.5 g, Sartorius), which follows the first, measures the weight of the drainage from the substrate bag. This system has been developed by the *Automation, Electronics, and Robotics Research Group at the University of Almería*.

The transpiration is calculated as the weight difference between two consecutive time-instants. The six plants scale is required for this calculation. Moreover, The two scales system, microlysimeter, allows for knowing when irrigation begins by changes in weight of the crop unit, as well as knowing when drainage starts (balance of drain) and when both end. As discussed above, an increase in the weight of the scale with the growing unit indicates that irrigation has begun. The process that follows is drainage warned by the heavy increase in the drainage scale, whose end would be indicated through the weight stabilization. From that time, the crop scale would start again to measure the weight loss (transpiration). During the process of irrigation drainage, the value of transpiration is considered as constant, taking the value of transpiration of the moment immediately preceding the irrigation beginning.

## 5. Results and Discussions

### 5.1. Transpiration Measurements Validation

**Figure 7.**
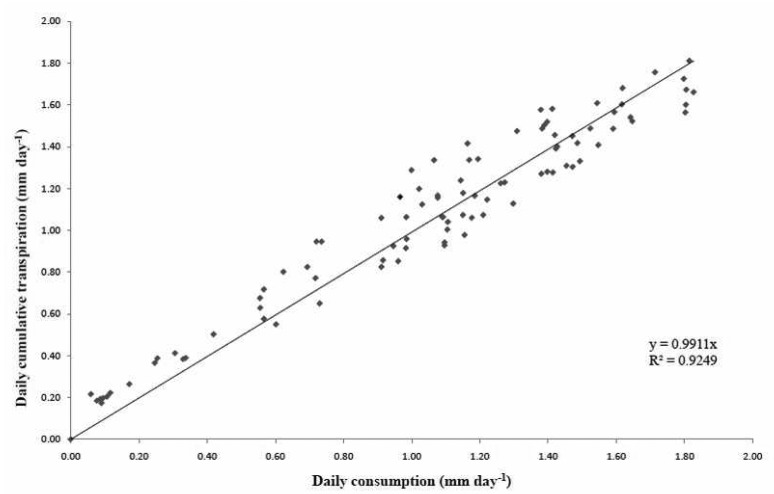
Testing the values of transpiration measured with daily consumption data in an autumn–winter cycle. It represents the daily accumulated transpiration and the daily average of the five trays with twelve plants consumption measurements.

**Figure 8.**
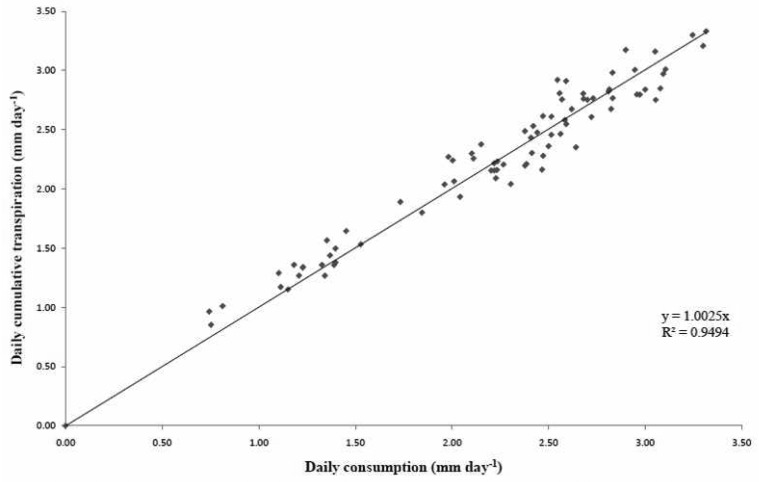
Testing the values of transpiration measured with daily consumption data in a spring-summer cycle. It represents the daily accumulated transpiration and the daily average of the five trays with twelve plants consumption measurements.

An important step was to validate the calculation of crop transpiration for each of the cycles, seeing that this data corresponds to the transpiration of the crop at that time. For this issue, five trays with twelve plants were installed and evenly distributed throughout the greenhouse so as to make their mean representative of the entire crop. The trays consisted of two bags of substrate with six plants in each bag, which gives us a total of twelve plants per tray and 60 in the entire test. All trays had drainage connected to a bucket whose sample was collected daily at the same hour. Thus, the average value of these buckets were taken daily to calculate the real drainage. Two differently located droppers were selected to collect daily irrigation amounts in the greenhouse; the final value was estimated from the average of both measures. With data from the drainage and from the droppers, the daily measured consumption was calculated and compared with accumulated daily transpiration from the data every minute, obtaining the graphs (Figures 7 and 8) for the two selected cycles (spring–summer 2008 and autumn–winter 2008–2009, respectively).

As shown in the graphs, daily and accumulated transpiration almost exactly match, according to *R*^2^ of the regression. In conclusion, transpiration data calculated from the balance system gives a closer idea of the values of real transpiration. Results of regression show a high R^2^ value and a slope with a value close to one, obtaining a graph of estimated and measured values approaching a 1:1 line, which would mean that for an instant ”*t*”, a value of transpiration very close to real plant consumption was obtained.

### 5.2. Virtual Sensors Calibration

Once the transpiration values were obtained, the next step was searching a substitution of this system with transpiration virtual sensors based on Penman–Monteith (P-M). The equation combines the energy balance with the vapor convective transport. In the last years, different physical and pseudo-physical virtual sensors (grey-box) based in the P-M equation have been developed and tested by different authors. For this paper, the pseudo-physical P-M simplification proposed by [2] was chosen because of the good results obtained by [6] for pepper crops in the same conditions. In this paper, these good results were obtained by fitting the parameters differentiating summer and winter seasons and with the different crop development stages. Furthermore, a delay is reported between measured and predicted values for some particular conditions. The causes of the observed delay were explored and a climate variables dependency was found as other authors assert ([6,29]). On the other hand, the proposed black-box dynamic virtual sensors would be used to design events based on fertirrigation controller. In order to use a modern control algorithm, the use of dynamic virtual sensors joined system identification techniques and are presented as an alternative to physical or pseudo-physical virtual sensors. The proposed virtual sensors incorporate the dynamics of transpiration and will be of varying complexity, beginning with linear black-box virtual sensors fitted to data. Nonlinear virtual sensors based in system identification was tried out obtaining good results. Nonlinearities will be introduced in an *ad hoc* form, relying on *a priori* knowledge about the system.

#### 5.2.1. Grey-Box Virtual Sensor

The calibration of the virtual sensor of transpiration proposed by [2] were performed with two different seasons: one in spring, in 2005 (Table 1), and the other in autumn–winter, in 2006–2007. For the calibration of the spring–summer cycle, all the data gathered during the months of February and July 2005 was used. In contrast, in the case of the winter cycle, the data used was from August 2006 to February 2007. The parameters were determined using an iterative sequential algorithm to minimize the least square error criterion between the real and the estimated transpiration (Montecarlo algorithms). The second phase of the calibration process was based in genetic algorithms to fix the final parameters. The values obtained from the extinction ratio of the radiation (*C_K_*), 0.64 for spring–summer cycles and 0.6 for autumn–winter, matched the results obtained by other authors in the autumn–winter cycle, with equivalent closeness, who obtained an extinction ratio of 0.63. [1] determined a value of 0.64 to cultivate the tomato, [6] obtained values of 0.63 to cultivate the cucumber. For most horticultural crops in greenhouses, the values of (*C_K_*) fluctuate between 0.4 and 0.8 [6]. Values obtained from parameters *C_A_*, *C_B_D__* and *C_B_N__* are different for both groups of crop cycles to which have been referred, obtaining values in the spring cycle for *C_A_*, *C_B_D__*, and *C_B_N__* of 0.49, 11.2, and 8.28 respectively, and for the autumn–winter cycle, the same parameters obtained values: *C_A_*, *C_B_D__*, and *C_B_N__* of 0.3, 18.7, and 8.3, respectively. The next table shows the results of the different parameters calibration:

In general, the values obtained from the ratio fell within the interval of values obtained by distinct authors, and gathered by [30]. A good behavior in the dynamic was observed in the virtual sensor, even though there is a small overestimation of the estimated values opposite those measured in the start and the end of the crop seasons. Even though this overestimation really exists, it does not reach a significant level, with which in the first analysis it could be concluded that correlation would not be necessary, though it is recommended. The presence of a phenomenon known as delay, which is translated to an advancement of the dynamics of the estimated values for real ones, was also demonstrated, as other authors have also previously asserted ([6,29]).

To calibrate grey-box virtual sensor described in section (Section 3.1), a growth model is required to try out the LAI estimations. The simplified model Tomgro [18] rises as an option to remove the complexity of the full virtual sensor proposed by [31] and make them available to online control systems while retaining their physiological characteristics [32]. The parameter that influence the dynamics of the *X_LAI_* was calibrated and validated, first by [18] and later by [33] in the same greenhouse for tomato crops.

#### 5.2.2. Linear Black-Box Virtual Sensors

To obtain a virtual sensor, it was necessary to choose two groups of transpiration data to try out in the system identification toolbox. One group from spring 2007 was taken for identification, resulting in a total of 53,490 data. For identification validation, 49,990 data from winter 2004 was used. The remaining data was used to obtain the virtual sensor's reliability. The black-box virtual sensor cannot contain data with time slots without data. For this reason, smaller groups of data are used. The Table 2 shows the virtual sensors that have been obtained. More than 1,500 structures were tested, leaving to validation two ARX virtual sensors and an ARMAX virtual sensor. In this case, LAI was not introduced into the system as an entry. The reason for this is that the rate of leaf area index remains constant in the same day, lacking the dynamic of remaining input variables. *X_LAI_* was used to divide the crop cycle in different intervals, from 0 to 0.7 =, from 0.7 to 1.5, and above 1.5 $\left(m_{\text{crop}}^{2}m_{\text{soil}}^{-2}\right)$. For the division, it is easy to change the LAI, for instance, by days after planting (DAP), or others time units.

The main problems encountered in the static virtual sensor are sought to be corrected by using dynamic virtual sensors, such as the overestimation that happens with low values of leaf area index and underestimation in very high values, as well as the presence of a phenomenon, which is translated in an advancement of the real dynamics over the estimated values.

#### 5.2.3. Nonlinear Black-Box Virtual Sensors

The first processing step was to eliminate the middle and both trends of inputs and outputs. It is worth noting that *X_LAI_* was not introduced into the system as an entry, because the leaf area index remains constant on the same day and lacks the dynamics as an input variable. To reach this conclusion, many tests with the Matlab toolbox were realized. First, *X_LAI_* was introduced in the virtual sensor as a regressor (*X_LAI_*(*t* − *i*)), obtaining a bad fix in the output. A regressor based on the equation of solar radiation reaching a given depth in canopy ([2]; Equation (6)) was tried out, but with the same results, such results was the expected:

(6) $$V_{G_{R}}e^{\left(-C_{k}X_{\text{LAI}}\right)}$$

where *V_G_R__* is the global radiation reaching the crop (*Wm* ^−2^), −*C_k_* is the extinction coefficient of radiation (unitless), and *X_LAI_* is the leaf area index $\left(m_{\text{crop}}^{2}m_{\text{soil}}^{-2}\right)$. As noted above, LAI remains constant during a chosen day as the parameter (*C_k_*), which means the regressor is constant during a day, depending exclusively on the radiation.

This virtual sensor was also evaluated by using an estimated VDP as a function of the inside temperature and the relative humidity. Despite this estimation, the virtual sensors obtained with these two variables show no improvement compared with those calibrated only using VPD. In the end, the radiation and the vapor pressure deficit remain as the unique inputs.

In order to obtain the number of terms in the regressors, it is necessary to obtain a model to try out different combinations. In addition to the number of terms obtained, the nonlinearities were estimated through different possibilities: wavelet network, tree partitions, and sigmoid network. Of all these tested ways to obtain the nonlinearities, the wavelet network gave the best fit joining a nonlinear block and a linear block. Table 3 shows the parameters of the resulting virtual sensor.

This virtual sensor was obtained with prediction aim.

### 5.3. Virtual Sensors Validation

**Figure 9.**
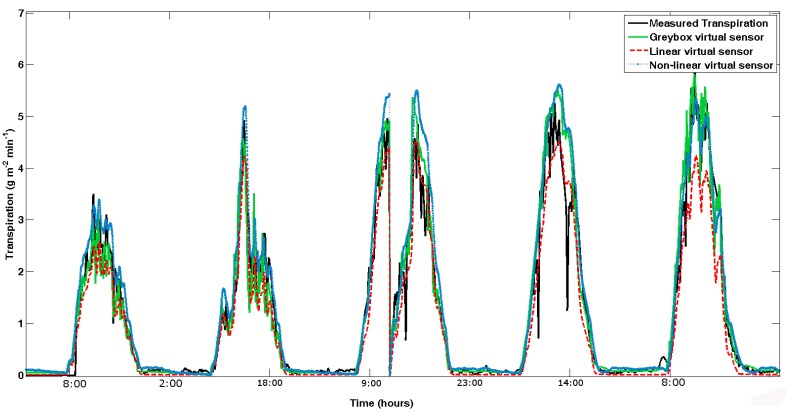
Five days in 2005 spring.

**Figure 10.**
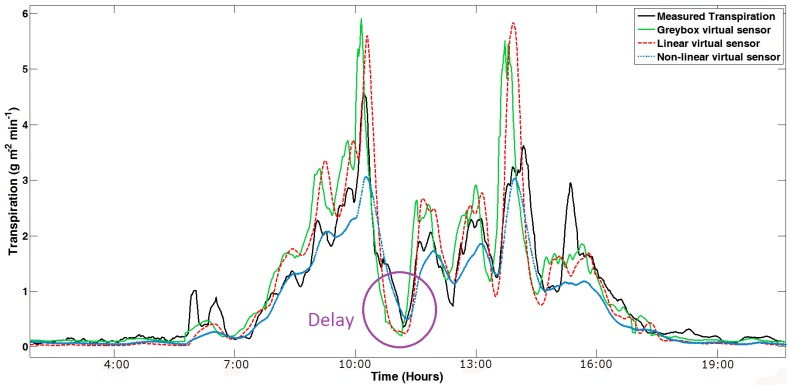
One full day with delay in the autumn–winter of 2007–2008. The figure starts at midnight.

**Figure 11.**
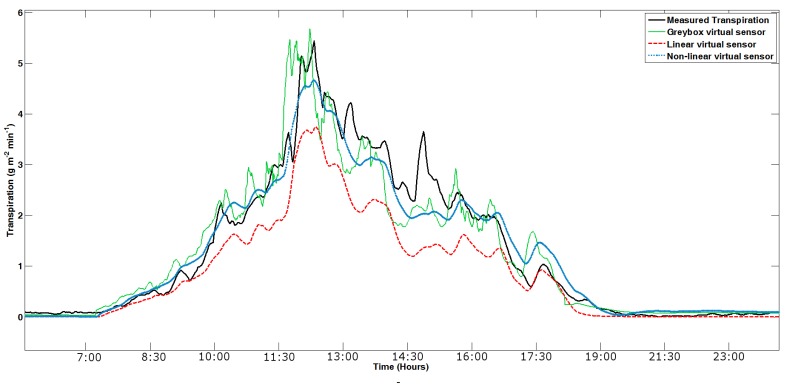
One full day in 2008 spring.

All available data (more than one million for each variable) has been used for the validation. In total, nine different spring–summer and autumn–winter seasons were used. Figures 9, 10 and 11 show an example of the results obtained in the validation process of the grey-box, as well as the linear and nonlinear dynamic virtual sensors in the different cycles. The validation of the virtual sensors can be seen in Figure 9. The presence of delay is demonstrated in Figure 10. In the end, Figure 11 samples a day detailed with the three virtual sensors. In some instances, the system dynamics is not well captured by the virtual sensors, as happens in Figure 10. This is caused by the difficulty in calculating the transpiration by using the microlysimeter. Furthermore, as Figure 9 shows, the transpiration behaves similarly to the sunlight: rising in the morning, lowering in the afternoon, and remaining almost constant at night.

The grey-box virtual sensor shows good dynamics. A small overestimation exists in the start and the end of the crop seasons but does not reach a significant level, with which in the first analysis it could be concluded that a correlation would not be necessary, although it is recommended. Furthermore, Figure 10 shows the delay phenomenon, which characterizes the advancement of the virtual sensor dynamics over the underlying physics, as other authors have asserted ([6,29]).

One of the characteristics of the calibration of linear and nonlinear black-box virtual sensor is that the first validation is done during the identification, since the trial and error processes must be done to choose the virtual sensor. Figures 9, 10 and 11 show that both black-boxes follow the dynamics of the tomato crop transpiration, presenting some adjustment problems in nocturnal areas. The problem of night setting is not of interest because it is sought. The night transpiration estimated through the virtual sensor obtains values higher that the pseudo-physical sensor. This overestimation is remarkable, but it is not very important for the final result, due to the fact that night transpiration measurements are very low in comparison with the daylight ones. The transpiration is so low, in fact, that it is difficult to take it with the microlysimeter, due to its 1 g sensor precision.

Moreover, this dynamic virtual sensor does not show the anticipation phenomenon, as is evident in the static virtual sensor. It is the resistance of the plant transpiration resulting in a delay of about the same processes that produce it, graphically shown as a delayed action on transpiration estimated.

**Figure 12.**
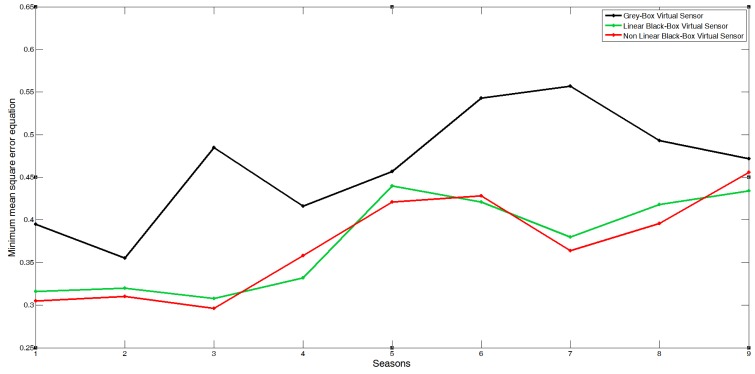
Virtual sensor error comparison, minimum mean square error in g m^−2^min^−1^.

For all the seasons included in this work (from the autumn in 2004 to the autumn in 2008–2009), the virtual sensor's goodness was obtained (the calibration seasons are marked with ∗ in the tables). This goodness for a data series is calculated through the minimum mean square error (MMSE). In all cases, the dynamic virtual sensor obtained good results (MMSE<6%), as can be seen in the three validation errors (see Table 4 and Figure 12).

Tables 5, 6 and 7 show a full review of the errors of each model.

## 6. Conclusions

Significantly, the research was conducted over three years, for a total of eight cycles of cultivation in those which took the different climatic and physiological variables of a tomato crop. All data were taken at intervals of one minute to give approximately over one million input data for each variable. The main objective, as noted at the beginning of this paper, is to implement the virtual transpiration sensor of tomato crops for the design of irrigation controllers. For this occasion, we had a measurement system based on transpiration weighing the difference that occurs on a scale from one moment to the next, and to measure water loss with a good approximation.

The next task was to seek a virtual sensor that would replace the system based on the weight difference caused by the loss of water by the crop. One was based on sensors that are typically installed in greenhouses: temperature, humidity, and solar radiation. The aim is to reduce total installation costs and to avoid the constant maintenance that the scales require.

After preliminary assessment of some of these virtual sensors, Nonlinear ARX had a better fit and, in the end, was the best election for the irrigation virtual sensor proposed. This virtual sensor had good results in the calibration and validation of the virtual sensor. An average error of 5%, for all cycles taken, shows how the choice of this virtual sensor was successful. However, it also presents some problems, such as the overestimation at night which occurs. On the other hand, linear and nonlinear black-box virtual sensors have demonstrated the absence of a advance phenomenon. It was translated to an advancement of the dynamics of the estimated values for real ones.

System identification techniques were chosen to obtain a dynamic virtual sensor. *Matlab*®software package was a good option to work with the identification techniques. A large number of different nonlinear dynamic virtual sensors structures was tested. The proposed dynamic for virtual sensors require only two inputs, global radiation and vapor pressure deficit, thus eliminating the inclusion of the *X_LAI_*. This virtual sensor presents the possibility of using modern control algorithms that cannot be used with the grey-box sensor.

As summary:

- Grey-box virtual sensor has a good fixing as advantage, but some disadvantages such as the overestimation at different moments of the year, a higher final error result, and the advance phenomenon.
- The black-box virtual sensors obtain better results and also allows the elimination of the grey-box problems: advance phenomenon and overestimation. An overestimation only appears during nocturnal periods.
- Nonlinear black-box had the best results.

This paper has dealt with the transpiration from an industrial point of view, as a process in which there are entries and exits. The crop itself, and some aspects of the climate inside the greenhouse, are considered disturbances affecting the dynamics. This shifts the focus away from the strictly agronomic, agronomy classic, which studies the exchanges that occur in the greenhouse by static virtual sensors based on fundamental principles, without including the system dynamic effect.

## Figures and Tables

**Table 1.** Results of the calibration in spring–summer and autumn–winter seasons.

Seasons	*C_A_*	*C_B_D__*	*C_B_N__*	*C_k_*
Autumn–winter	0.3	18.7	8.5	0.6
Spring–summer	0.49	11.2	8.28	0.64

**Table 2.** Results of the calibration in spring–summer and autumn–winter seasons. Where LAI is the Leaf Area Index (dimensionless); *na* is the parametric equation's order that defines the outputs; *nb* is the order of the input; *nc* is the error order; and *nk* the delay of the radiation solar (*nk_V_G_R___*) and the vapor pressure deficit (*nk_V _V PD__*).

LAI interval	Virtual sensor	na	nb	nc	nk^V_G_R__^	nk^V _V PD_^
0.7 or lower	ARX450	4	5		0	0
0.7 to 1.5	ARX540	5	4		0	0
1.5 or higher	ARMAX55240	5	5	2	4	0

**Table 3.** Results of the nonlinear virtual sensor calibration: *na* is the parametric equation's order of outputs; *nb* is the order of the input; and *nk* is the delay of the solar radiation (*nk_V_G_R___*) and of the vapor pressure deficit (*nk_V_V PD__*).

Virtual sensor	na	nb	*nk_V_G_R___*	*nk_V_V PD__*
NonARX2230	2	2	4	1
NonARX4430	4	4	4	1

**Table 4.** Virtual sensor error comparison, in g m^−2^min^−1^.

Season	Samples	Grey-box	Linear Black-box	Nonlinear Black-box	E interval
A-w 2004–2005	104, 831	0.395	0.316	0.305	[0, 9.40]
S-s 2005 ∗	125, 704	0.355	0.320	0.310	[0, 12.87]
A-w 2005–2006	164, 790	0.485	0.308	0.296	[0, 9.60]
S-s 2006	89, 000	0.416	0.332	0.358	[0, 10.27]
A-w 2006–2007	104, 817	0.457	0.440	0.421	[010.73]
S-s 2006	149, 807	0.543	0.421	0.428	[0, 11.33]
A-w 2007–2008	123, 657	0.557	0.380	0.364	[0, 10.30]
S-s 2008	132, 654	0.493	0.418	0.396	[0, 11.90]
A-w 2008–2009	106, 855	0.472	0.434	0.456	[0, 10.45]

**Table 5.** Grey-virtual sensor error calculation in g m^−2^min^−1^.

Season	Samples	Max error	Mean Error	σ	λE interval
A-w 2004–2005	104, 831	2.12	0.395	0.491	[0, 9.40]
S-s 2005 ∗	125, 704	3.65	0.355	0.500	[0, 12.87]
A-w 2005–2006	164, 790	2.79	0.485	0.740	[0, 9.60]
S-s 2006	89, 000	3.01	0.416	0.747	[0, 10.27]
A-w 2006–2007	104, 817	2.86	0.457	0.598	[0, 10.73]
S-s 2007	149, 807	3.28	0.543	0.812	[0, 11.33]
A-w 2007–2008	123, 657	2.92	0.557	0.602	[0, 10.30]
S-s 2008	132, 654	3.44	0.493	0.612	[0, 11.90]
A-w 2008–2009	106, 855	3.16	0.472	0.581	[0, 10.45]

**Table 6.** Empirical (black-box) virtual sensors error calculation in g m^−2^min^−1^.

Season	Samples	Max error	Mean Error	σ	λE interval
A-w 2004–2005	104, 831	1.42	0.305	0.398	[0, 9.40]
S-s 2005 ∗	125, 704	2.56	0.310	0.406	[0, 12.87]
A-w 2005–2006	164, 790	2.41	0.296	0.392	[0, 9.60]
S-s 2006	89, 000	2.21	0.358	0.607	[0, 10.27]
A-w 2006–2007	104, 817	2.75	0.421	0.576	[010.73]
S-s 2006	149, 807	1.98	0.428	0.639	[0, 11.33]
A-w 2007–2008	123, 657	2.02	0.364	0.523	[0, 10.30]
S-s 2008	132, 654	2.21	0.396	0.498	[0, 11.90]
A-w 2008–2009	106, 855	2.69	0.434	0.512	[0, 10.45]

**Table 7.** Nonlinear virtual sensor minimum mean square error in g m^−2^min^−1^.

Season	Samples	Max error	Mean Error	σ	λE interval
A-w 2004–2005	104, 831	1.21	0.316	0.387	[0, 9.40]
S-s 2005 ∗	125, 704	2.33	0.320	0.407	[0, 12.87]
A-w 2005–2006	164, 790	2.19	0.308	0.410	[0, 9.60]
S-s 2006	89, 000	2.42	0.332	0.601	[0, 10.27]
A-w 2006–2007	104, 817	2.75	0.440	0.579	[010.73]
S-s 2006	149, 807	1.96	0.421	0.657	[0, 11.33]
A-w 2007–2008	123, 657	2.02	0.380	0.539	[0, 10.30]
S-s 2008	132, 654	2.20	0.418	0.496	[0, 11.90]
A-w 2008–2009	106, 855	2.40	0.456	0.519	[0, 10.45]
